# Urinary peptide analysis to predict the response to blood pressure medication

**DOI:** 10.1093/ndt/gfad223

**Published:** 2023-10-31

**Authors:** Mayra Alejandra Jaimes Campos, Emmanouil Mavrogeorgis, Agnieszka Latosinska, Susanne Eder, Lukas Buchwinkler, Harald Mischak, Justyna Siwy, Peter Rossing, Gert Mayer, Joachim Jankowski

**Affiliations:** Mosaiques Diagnostics GmbH, Hannover, Germany; University Hospital RWTH Aachen, Institute for Molecular Cardiovascular Research, Aachen, Germany; Mosaiques Diagnostics GmbH, Hannover, Germany; University Hospital RWTH Aachen, Institute for Molecular Cardiovascular Research, Aachen, Germany; Mosaiques Diagnostics GmbH, Hannover, Germany; Department of Internal Medicine IV (Nephrology and Hypertension), Medical University Innsbruck, Innsbruck, Austria; Department of Internal Medicine IV (Nephrology and Hypertension), Medical University Innsbruck, Innsbruck, Austria; Mosaiques Diagnostics GmbH, Hannover, Germany; Mosaiques Diagnostics GmbH, Hannover, Germany; Steno Diabetes Center Copenhagen, Complications Research, Copenhagen, Denmark; Department of Clinical Medicine, University of Copenhagen, Copenhagen, Denmark; Department of Internal Medicine IV (Nephrology and Hypertension), Medical University Innsbruck, Innsbruck, Austria; Department of Internal Medicine IV (Nephrology and Hypertension), Medical University Innsbruck, Innsbruck, Austria; Department of Pathology, Cardiovascular Research Institute Maastricht (CARIM), University of Maastricht, Maastricht, The Netherlands; Aachen-Maastricht Institute for Cardiorenal Disease (AMICARE), University Hospital RWTH Aachen, Aachen, Germany

**Keywords:** biomarkers, CKD, progression, type 2 diabetes, urine proteomics

## Abstract

**Background:**

The risk of diabetic kidney disease (DKD) progression is significant despite treatment with renin–angiotensin system (RAS) blocking agents. Current clinical tools cannot predict whether or not patients will respond to treatment with RAS inhibitors (RASi). We aimed to investigate whether proteome analysis could identify urinary peptides as biomarkers that could predict the response to angiotensin-converting enzyme inhibitor and angiotensin-receptor blockers treatment to avoid DKD progression. Furthermore, we investigated the comparability of the estimated glomerular filtration rate (eGFR), calculated using four different GFR equations, for DKD progression.

**Methods:**

We evaluated urine samples from a discovery cohort of 199 diabetic patients treated with RASi. DKD progression was defined based on eGFR percentage slope results between visits (∼1 year) and for the entire period (∼3 years) based on the eGFR values of each GFR equation. Urine samples were analysed using capillary electrophoresis–coupled mass spectrometry. Statistical analysis was performed between the uncontrolled (patients who did not respond to RASi treatment) and controlled kidney function groups (patients who responded to the RASi treatment). Peptides were combined in a support vector machine-based model. The area under the receiver operating characteristic curve was used to evaluate the risk prediction models in two independent validation cohorts treated with RASi.

**Results:**

The classification of patients into uncontrolled and controlled kidney function varies depending on the GFR equation used, despite the same sample set. We identified 227 peptides showing nominal significant difference and consistent fold changes between uncontrolled and controlled patients in at least three methods of eGFR calculation. These included fragments of collagens, alpha-1-antitrypsin, antithrombin-III, CD99 antigen and uromodulin. A model based on 189 of 227 peptides (DKDp189) showed a significant prediction of non-response to the treatment/DKD progression in two independent cohorts.

**Conclusions:**

The DKDp189 model demonstrates potential as a predictive tool for guiding treatment with RASi in diabetic patients.

KEY LEARNING POINTS **What was known:**

- Identifying diabetic patients at risk of progressive kidney function loss despite renin–angiotensin system inhibitor (RASi) therapy is crucial for clinical decision-making.
- The utility of estimated glomerular filtration rate (eGFR) equations based on serum creatinine, cystatin C or their combination for predicting diabetic kidney disease (DKD) progression remains uncertain.
- Non-invasive biomarkers are needed to predict DKD progression and response to therapy.

**This study adds:**

- We demonstrate significant association of multiple urinary peptides with DKD progression under RASi treatment.
- Introduction of a novel urinary peptide-based classifier for predicting response to RASi treatment.
- Variability in patient labelling as uncontrolled/controlled kidney function based on different eGFR calculation methods highlights the importance of universally and accurate approaches.

**Potential impact:**

- Urinary peptides may inform about response to RASi treatment.
- The application of this classifier shows promise in identifying diabetic patients likely to respond to RASi treatment, improving DKD management strategies.

## INTRODUCTION

Chronic kidney disease (CKD) is a major global public health problem characterized by the progressive loss of kidney function or structure [1]. Diabetic kidney disease (DKD) has been identified as a primary cause of increased mortality risk in patients with diabetes [2, 3]. Renin-angiotensin system (RAS) blockers may, to varying degrees, help delay the progression of CKD, particularly in DKD [4, 5]. However, while angiotensin-receptor II blockers (ARBs) are generally effective and rapid in achieving their primary goal of reducing blood pressure, assessing their impact on CKD progression requires a longer period of observation and cannot be assessed promptly. Indeed, they are not designed for the treatment of CKD.

Reports have shown promising results of capillary electrophoresis–coupled mass spectrometry (CE-MS)-based urinary proteome analysis in the context of early disease detection, prognosis of progression and therapy response assessment in different diseases [6–11]. In the context of CKD, the urinary peptide-based classifier CKD273 has demonstrated its capability to effectively classify patients with varying renal function and accurately predict early CKD progression in independent cohorts [8, 12]. Moreover, in individuals with type 2 diabetes mellitus (T2DM) and albuminuria, reduced levels of CKD273 classifier have been observed after treatment, further emphasizing its potential clinical significance [6].

The Kidney Disease: Improving Global Outcomes (KDIGO) 2012 guidelines stratify CKD and the risk of progression based on glomerular filtration rate (GFR) and albuminuria markers [13]. In clinical practice, direct measurement of GFR is often not feasible. Instead, GFR is estimated using different equations that include serum creatinine (eGFRcr), cystatin C (eGFRcys) levels or the combination of both (eGFRcr-cys), as well as additional demographic parameters like age, race and sex [14]. The accuracy of eGFR estimation based on creatinine levels can be suboptimal due to various factors [15]. As a result, alternative equations based on cystatin C levels have been developed to improve the accuracy of eGFR estimates [15]. In addition to accuracy, the prognostic value of these equations is also clinically important and has been evaluated in patients with cardiovascular pathologies and acute kidney injury [16–18]. To the best of our knowledge, no study has compared the utility of these equations to support the prediction of CKD development in diabetic patients under anti-hypertensive treatment. At the same time, the availability of biomarkers that can predict early progression of DKD and response to therapy would be crucial in guiding intervention.

Based on these facts and clinical needs, we aimed to investigate the potential of CE-MS-identified urinary peptide biomarkers in guiding intervention in DKD progression. Specifically, we wished to determine whether peptides could support prediction of progression in DKD despite RASi treatment like angiotensin-converting enzyme inhibitor (ACEi) and ARBs (uncontrolled), contrasted by stabilizing kidney function (controlled). As a further aspect of the study, we aimed to explore the validity of assessment of eGFR percentage slope based on a ∼1-year compared with a 3-year follow-up. To ensure the correct labeling of patients as having uncontrolled or controlled kidney function, we initially evaluated the concordance between four different eGFR equations.

## MATERIALS AND METHODS

### Study participants

This study evaluated three different cohorts of adults, comprising one discovery cohort (DC-REN: Drug combinations for rewriting trajectories of renal pathologies in type II diabetes) [19] and two validation cohorts (PRIORITY: Proteomic prediction and renin angiotensin aldosterone system inhibition prevention of early diabetic nephropathy in type 2 diabetic patients with normoalbuminuria [7] and DIRECT-Protect 2: Diabetic Retinopathy Candesartan Trials) [20, 21]. DC-REN cohort is part of a prospective study (PROVALID) performed in 4000 patients in five European countries to validate biomarkers in people with T2DM [19]. For the discovery study in DC-REN, all individuals treated with RASi with a total of three or four urine samples available and collected in yearly intervals (*n* = 199) were included. To calculate the eGFR values in DC-REN cohort, the CKD Epidemiology Collaboration (CKD-EPI) formulas for creatinine, cystatin or a combination of both [22], as well as The European Kidney Function Consortium (EKFC) equation for cystatin C [15], were used.

PRIORITY [7] was a prospective, international multicentre study of 1775 participants with T2DM treated with spironolactone or placebo. Patients treated with ARB and ACEi and other anti-hypertensive drugs (but not treated with spironolactone) were selected (*n* = 1078). They were then categorized based on controlled or uncontrolled kidney function, resulting in a sample size of *n* = 468. DIRECT-Protec 2 [20, 21] was a prospective cohort study of 1905 individuals with T2DM of whom 951 were treated with candesartan. For this study, we selected all patients with a urine sample available and who underwent candesartan treatment (*n* = 365). They were subsequently categorized based on their kidney function being controlled or uncontrolled, resulting in a total sample size of *n* = 194. To assess whether the prediction of uncontrolled kidney function in the context of RASi treatment is specifically associated with the treatment, we also investigated patients receiving a placebo in the DIRECT-Protec 2 cohort, a total of 216 individuals (uncontrolled *n* = 106, controlled *n* = 110).

The estimation of eGFR values was based on the CKD-EPI creatinine equation for both validation cohorts. Information regarding the studies, their designs and the methods used has been published previously [17, 20–22]. All individuals included in this study signed written informed consents and the protocols followed the ethical standards per the Helsinki Declaration. The PROVALID study protocol was approved in each participating country by the responsible local Institutional Review Board. This study was approved by the Medical University Innsbruck ethics committee under the reference number EK 1188/2020.

### Study design and progression definition

All patients in the discovery cohort had a baseline visit and at least two follow-up visits after collecting the urine samples. The mean number of follow-ups per patient was 3.93 ± 0.37, 2.50 ± 0.84 and 4.44 ± 0.86 in DC-REN, PRIORITY and DIRECT-Protec 2 cohorts, respectively. At baseline and follow-up, values for eGFR (calculated with the different formulas in DC-REN), age, gender, duration of diabetes diagnosis, blood pressure, weight, body mass index (BMI) and history of cardiovascular diseases values were obtained. The eGFR slope during follow-up was calculated by fitting a straight line through the longitudinally measured eGFR values using linear regression with the ‘lm’ function in R software (R version 4.1.0, R Foundation for Statistical Computing, Vienna, Austria). Specifically, the model included eGFR as the dependent variable and follow-up time as the independent variable, providing regression coefficients and their corresponding *P*-values. In the DC-REN cohort, eGFR percentage slope between visits (mean of 1 ± 0.56 year) and for the entire period, based on the eGFR values of each equation, were calculated. Patients with a reduction of more than 10% of eGFR per year were defined as uncontrolled, e.g. not responding to RASi treatment. On the other hand, patients with eGFR percentage slope values between +5% and –5% were assigned to the controlled kidney function group, e.g. responding to the RASi treatment. Individuals losing between 5% and 10% or who gained more than 5% were excluded as they could not be labelled with confidence as controlled or uncontrolled. This decision is consistent with previous studies showing that patients with improved renal function (>5% eGFR percentage decline) have a significantly different peptidomic profile compared with controlled patients [23].

The patient status (controlled or uncontrolled) was defined in the validation cohorts based on stricter criteria. Patients with a reduction of >10 mL/min/1.73 m^2^ in eGFR from baseline during follow-up and additionally progressing to DKD stage 3 (eGFR value <60 mL/min/1.73 m^2^) were defined as having uncontrolled kidney function, patients losing <5 mL/min/1.73 m^2^ in PRIORITY or <10 mL/min/1.73 m^2^ in DIRECT-Protec 2 over the entire study period and with eGFR always >60 mL/min/1.73 m^2^ were labelled as controlled kidney function.

### Proteome profiles

Proteome analysis was performed as described previously [24]. Briefly, the urine samples were thawed immediately before use and diluted with 2 M urea, 10 mM NH_4_OH containing 0.02% SDS. Subsequently, samples were filtered and desalted using PD-10 column (GE Healthcare, Sweden) and equilibrated in 0.01% NH_4_OH. Finally, filtrate was lyophilized and stored at 4°C until CE-MS analyses. The proteome profiles followed the standard operating procedures and quality control criteria described previously [24]. Mass spectral peaks were deconvoluted using MosaFinder software [25]. Normalization was done using a linear regression algorithm with internal standard peptides as reference [26]. Finally, detected peptides were deposited, matched and annotated in a Microsoft SQL database. For identification of the amino acid sequences corresponding to the peptides detected by CE-MS, the acquired masses were compared with peptide sequences derived from liquid chromatography–tandem mass spectrometry (LC-MS/MS) analysis using LTQ Orbitrap hybrid MS (Thermo Fisher Scientific, Bremen, Germany) with a nano-electrospray ion source. Data files were searched against the UniProt human non-redundant database using Proteome Discoverer 2.4 and the SEQUEST search engine, without enzyme specification (activation type: HCD; precursor mass tolerance: 5 p.p.m.; fragment mass tolerance: 0.05 Da) [27].

### Statistical analysis and generation of the model

The CE-MS datasets of the DC-REN cohort were used to define potential biomarkers. The statistical comparison between uncontrolled and controlled patients was conducted using a peptide frequency threshold of 30% in at least one group. *P*-values were calculated using a Wilcoxon test with a ‘two-sided’ alternative hypothesis with the R package ‘wilcox.test’ and the fold change was defined as the mean of the uncontrolled group divided by the mean of the controlled group. The association between eGFR percentage slope and the peptidomics data was analysed using Spearman's rank correlation with the package ‘cor_test’. To assess the performance of the different eGFR equations, linear regression analyses were conducted using the package ‘lm’. Analyses were performed using R (R version 4.1.0, R Foundation for Statistical Computing, Vienna, Austria).

To develop the model, we selected peptides that exhibited a significant nominal difference (Wilcoxon test *P*-value <.05) and consistent fold changes between uncontrolled and controlled patients in at least three methods of eGFR calculation. The EKFC eGFRcys dataset was used to develop the model using a support vector machine (SVM) algorithm integrated into the MosaCluster software [28] and for further analysis. MedCalc software was used to determine the area under the receiver operating characteristic (ROC) curve (AUC) of the generated model and the graphs were done with the R package ‘ggplot2’.

## RESULTS

### Clinical characteristics and patients classification

**Figure 1:**
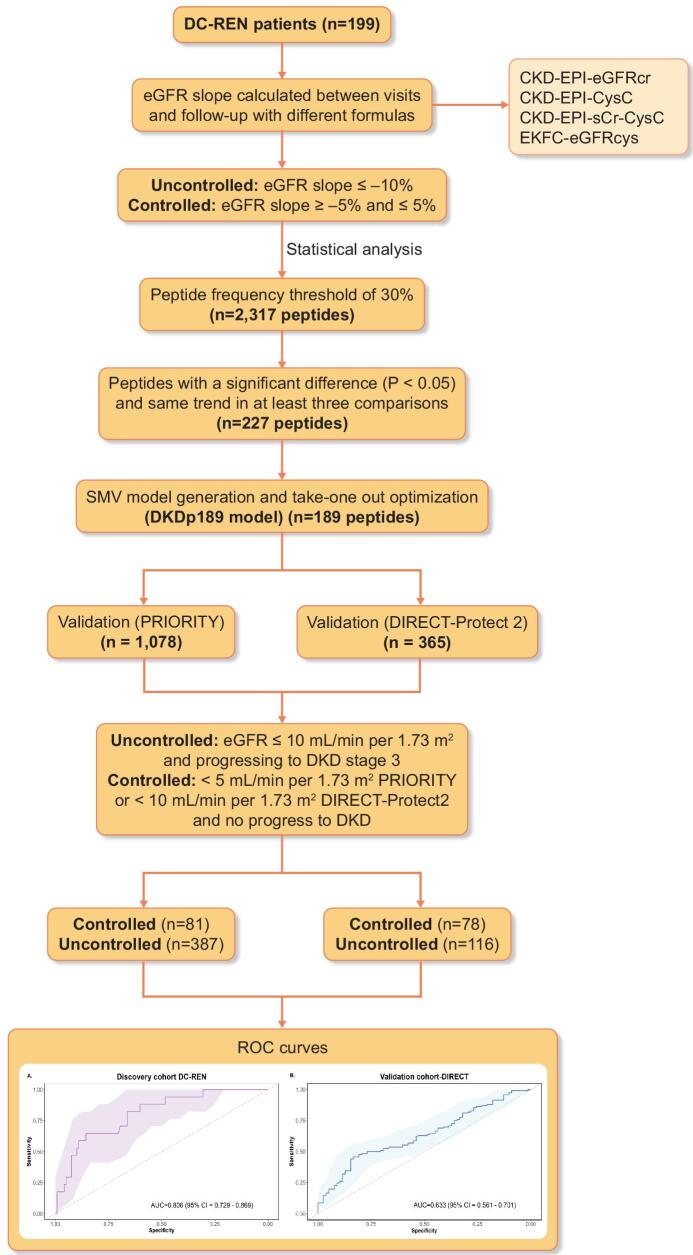
Study design and workflow for the selection of the biomarkers. The different methods were used to identify the CKD progression associated peptides. Biomarkers were defined based on EKFC eGFRcys category using a support vector machine model. Two independent cohorts were used for the validation (PRIORITY and DIRECT-Protec 2).

The study design is graphically depicted in Fig. 1, while the characteristics of the study populations at baseline are detailed in Table 1. Regarding the discovery cohort, the baseline mean age was 67.5 years. Slightly more than half of the population were females, with a percentage of 53.3%. The average time since the diagnosis of T2DM was 13.4 years. The baseline means eGFR calculated with CKD-EPI eGFRcr was 63.30 mL/min/1.73 m^2^ and was not significantly different from the eGFR calculated using other equations (Kruskal–Wallis rank test, *P*-value = .3916). Mean weight and BMI were 85.9 kg and 30.6 kg/m^2^, respectively. The mean HbA1c level among the participants was 7.1%. Additionally, 30.2% of the population had a history of cardiovascular disease. The means of systolic and diastolic blood pressure were 137.7 mmHg and 77.7 mmHg, respectively.

**Table 1:** Baseline characteristics of the cohorts.

	DC-REN, *n* = 199	PRIORITY, *n* = 468	DIRECT-Protec-2, *n* = 194
Mean follow-up per patient, years	3.93 (0.37)	2.50 (0.84)	4.44 (0.86)
Age, years	67.54 (8.04)	63.10 (7.41)	56.72 (7.10)
Gender, *n* (%)			
Male	93 (46.73)	308 (65.81)	105 (54.12)
Female	106 (53.27)	160 (34.19)	89 (45.88)
Duration of diabetes, years	13.42 (8.19)	12.59 (7.89)	9.11 (5.00)
CKD stage 3 at baseline (eGFR value <60 mL/min/1.73 m^2^), *n* (%)	78 (39.20)	0 (0)	1 (0.56)
eGFR, mean (SD), mL/min/1.73 m^2^			
CKD-EPI eGFRcr	63.30 (16.77)	85.42 (12.91)	71.20 (7.19)
CKD-EPI eGFRcys	61.86 (19.59)	NA	NA
CKD-EPI eGFRcr-cys	63.51 (18.19)	NA	NA
EKFC eGFRcys	63.28 (16.62)	NA	NA
Weight, kg	85.94 (16.35)	NA	83.74 (15.03)
BMI, kg/m^2^	30.60 (4.69)	30.56 (4.66)	29.54 (4.78)
HbA1c, %	7.07 (1.09)	NA	8.07 (1.57)
History of cardiovascular disease, *n* (%)	60 (30.15)	NA	9 (4.64)
Systolic blood pressure, mmHg	137.72 (16.65)	134.23 (12.00)	NA
Diastolic blood pressure, mmHg	77.73 (10.20)	78.61 (9.14)	NA

Data are mean [standard deviation (SD)] or *n* (%).

NA, not available; Cr, serum creatinine; HbA1c, hemoglobin A1c.

**Figure 2:**
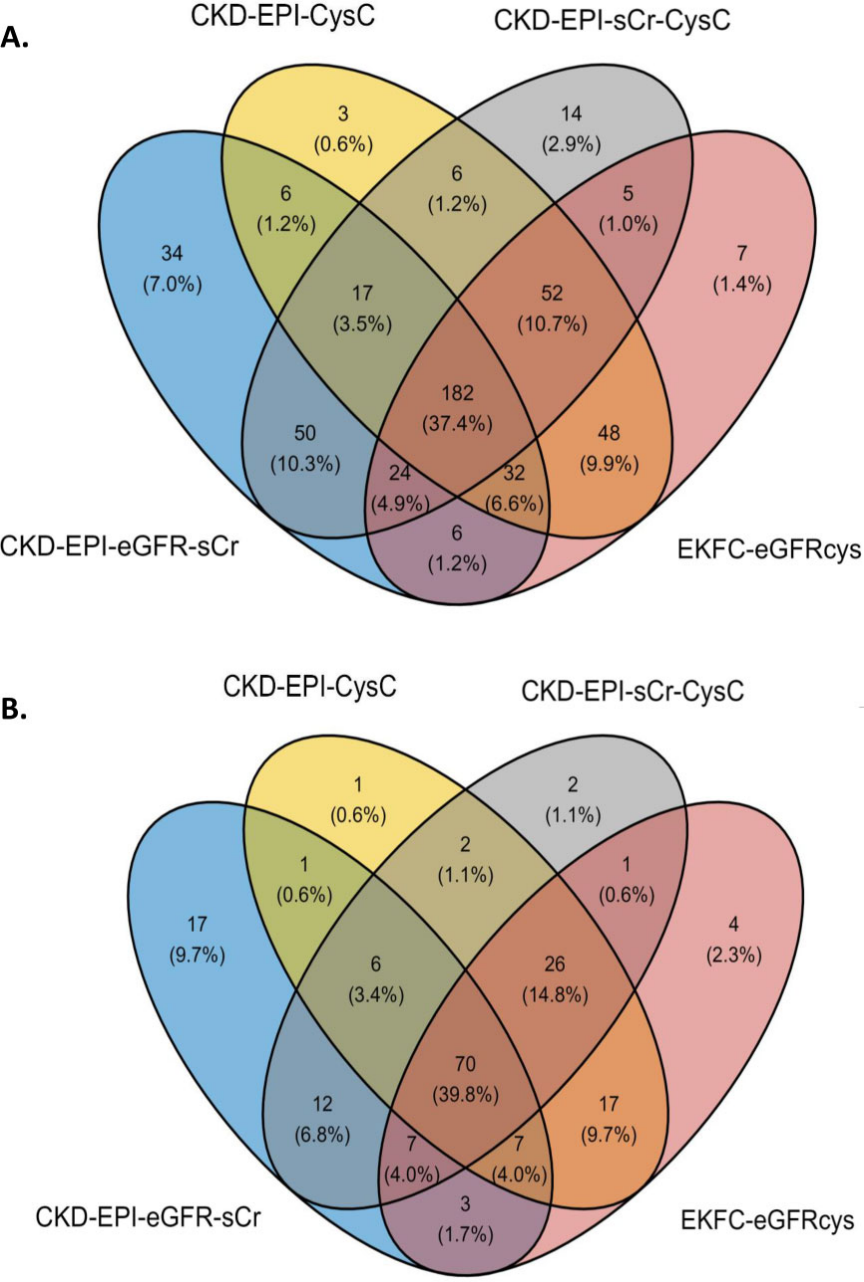
Comparison of classification between uncontrolled and controlled patients of the CKD-EPI and EKFC equations (**A**) between visits (1 year) and (**B**) for the entire period (3 years).

**Figure 3:**
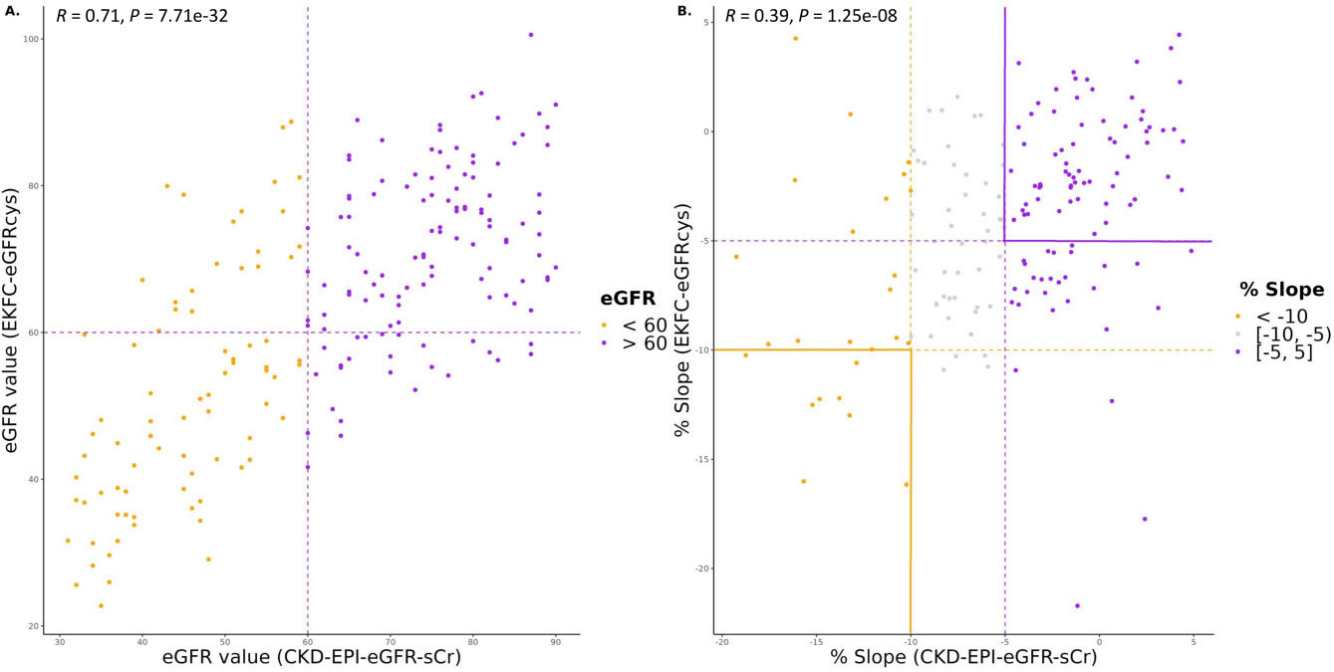
Association of baseline eGFR values and percentage slope during follow-up calculated with CKD-EPI eGFRcr and EKFC eGFRcys equations. (**A**) Scatter diagram for correlation of baseline eGFR values calculated with CKD-EPI eGFRcr and EKFC eGFRcys equations. (**B**) Scatter diagram for correlation of percentage slope values during follow-up calculated with CKD-EPI eGFRcr and EKFC eGFRcys equations.

Labelling kidney function as uncontrolled or controlled was based on the eGFR percentage slopes, calculated as described in the Materials and methods section. For each eGFR calculation method the classification was performed separately. The concordance of the uncontrolled and controlled labels between the four different eGFR calculation methods is shown in Fig. 2. The distribution of uncontrolled and controlled patients varies depending on the equation used, despite the same sample set. The 3-year period–based percentage slope results have a slightly higher overlap among methods (39.8%, Fig. 2B) than between visits-based percentage slope results (37.4%, Fig. 2A). When comparing the distribution in the baseline eGFR values between CKD-EPI eGFRcr and EKFC eGFRcys equations, we observed a high correlation (r = 0.71, *P* = 7.71e-32) (Fig. 3A). However, when examining the correlation between the percentage slope values during follow-up between the same equations, we observed a substantially reduced agreement (r = 0.39, *P* = 1.25e-08) (Fig. 3B) and this trend continued when all equations were compared (Supplementary data, Fig. S1). Furthermore, we performed a correlation analysis to assess the association between peptides identified and the percentage slope eGFR values at 3 years. To compare the performance of the different eGFR equations, we conducted a linear regression analysis, selecting rho values with a *P*-value <.1 (Supplementary data, Table S1). Remarkably, the CKD-EPI eGFRcr equation exhibited a weaker correlation (r^2^ between 0.48 and 0.68) compared with the other formulas, where r^2^ was consistently >0.8.

### Identification of biomarkers and validation analyses

**Figure 4:**
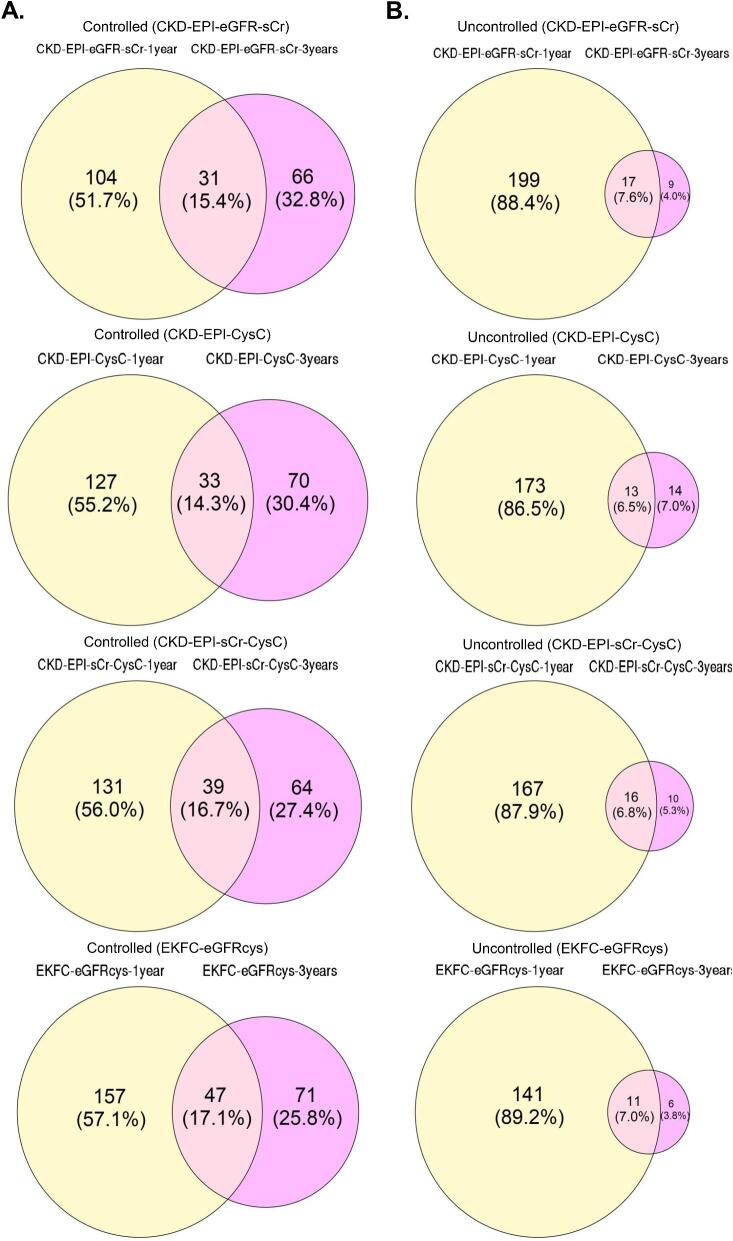
Venn diagrams for overlap of controlled (**A**) and uncontrolled (**B**) patients at ∼1 year follow-up and 3-year follow-up with the different equations.

In the DC-REN cohort, 2317 peptides were observed at a frequency above 30% in at least one group. After separating the entire cohort into sets of uncontrolled and controlled patients, based on the four different equations to estimate GFR, statistical evaluation to identify potential biomarkers was performed for each method separately (Supplementary data, Table S2). The available sample size did not allow adjustment for multiple testing in most experiments, and consequently peptides of nominal significance were selected as potential biomarkers. As evident from Supplementary data, Table S2 and summarized in Table S4, the overlap of significant peptides (Wilcoxon *P*-value <.05) found by each method of eGFR assessment was moderate. A large number of peptides reached nominal significance only in one approach. To examine which method resulted in the highest concordance, when compared with the independent PRIORITY and DIRECT-Protec 2 cohorts, we compared the statistical analysis results (per method) with the respective ones in the discovery cohort. Specifically, we assessed the number of peptides with either a *P*-value <.05 in the discovery cohort and the same regulation trend or only considering the same regulation trend (Supplementary data, Tables S2 and S4). Based on this comparison, the EKFC eGFRcys equation had the highest number of peptides verified in PRIORITY and DIRECT-Protec 2 cohorts (6.12% at ∼3-year follow up) compared with the other methods (0.88%–3%). As expected, the results from the 3-year follow-up in all equations appeared to be more stable to random variation compared with the ∼1-year follow-up (28.85% ± 3.12 vs 54.92% ± 2.33 and 4.74% ± 1.48 vs 87.78% ± 1.23, in uncontrolled and controlled patients, respectively). This is likely due to the longer period analysed and the increased number of eGFR assessments per patient, which was expected to result in increased accuracy and being less affected by short-term fluctuations (Fig. 4).

### Prediction of non-response to RASi treatment (DKD progression)

**Figure 5:**
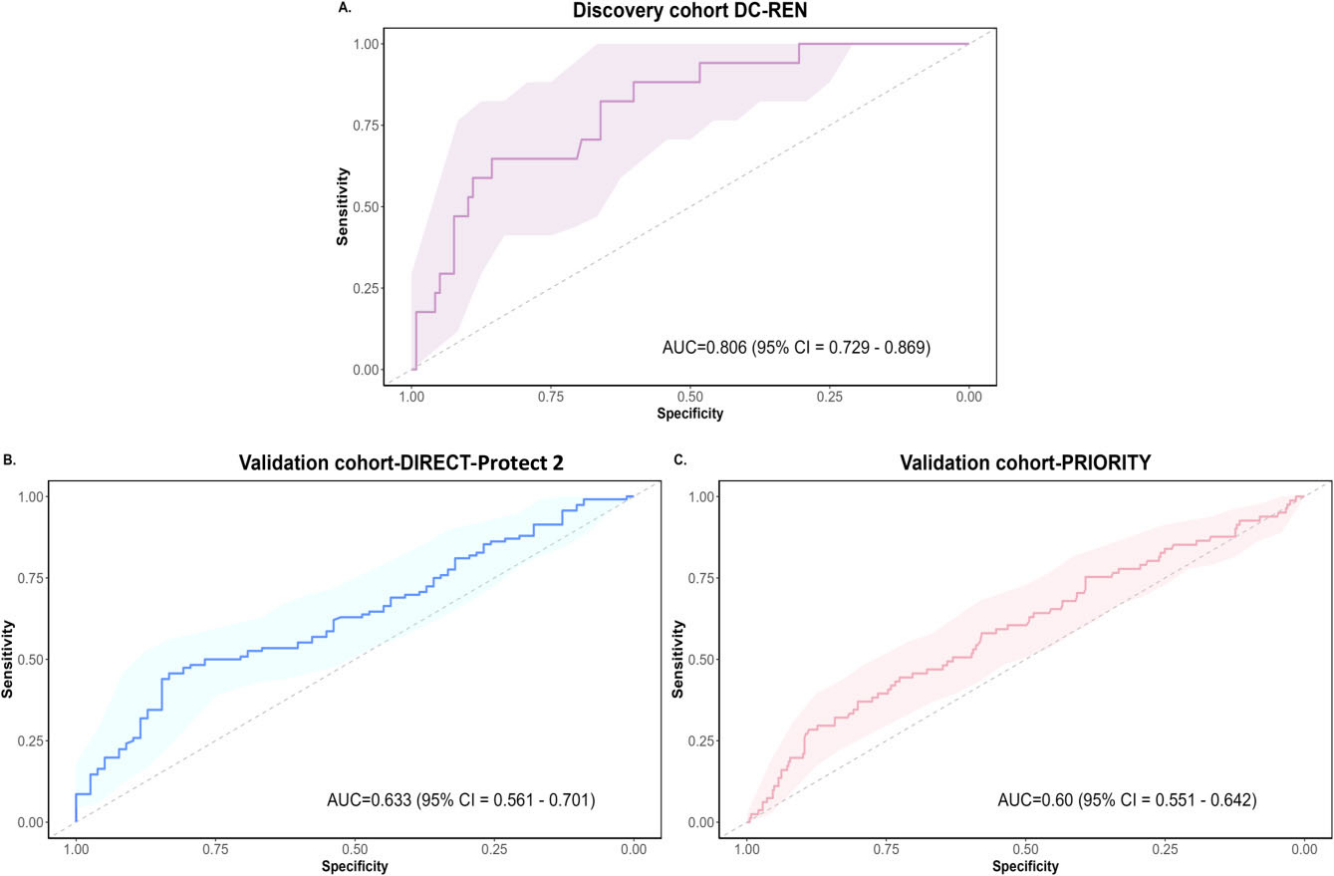
ROC curves for the DKDp189 model. (**A**) n-1 cross/validated training ROC curve in the discovery cohort (DC-REN) using the EKFC eGFRcys dataset. (**B**) ROC curve in the DIRECT-Protec 2 validation cohort. (**C**) ROC curve in the PRIORITY validation cohort.

As a result of the inconsistencies in assessing DKD progression and since none of the methods could be used as a ‘gold standard’, we decided to define potential biomarkers most consistent in the different assessments. We identified a total of 227 peptides showing a nominal significant difference (Wilcoxon test <0.05) and the same directional change (up- or down-regulated) in at least three of the four eGFR calculation methods. Most of the peptides that showed a significant association between the methods are specific fragments of collagen, predominantly derived from collagen type I. Fragments of alpha-1 antitrypsin, antithrombin, CD99 antigen, uromodulin and alpha-2-HS-glycoprotein were also detected. We combined these biomarkers to create a SVM-based model and optimized it using a take-one-out procedure. This process yielded a model based on 189 peptides (DKDp189 model, C = 256, gamma = 2e-005). The n-1 cross-validated discovery cohort ROC yielded an AUC of 0.806 [95% confidence interval (CI) 0.729–0.869, *P*-value <.0001] (Fig. 5A). To validate the results, we applied the model to two independent cohorts of diabetes patients treated with RASi and investigated model's performance to predict the response to the treatment. The model significantly separated controlled from uncontrolled patients with an AUC of 0.633 (95% CI 0.561–0.701, *P*-value = .0008) in DIRECT-Protec 2 (Fig. 5B) and 0.60 (95% CI 0.551–0.642, *P*-value = .0074) in the PRIORITY cohort (Fig. 5C).

Additionally, to assess the predictive capacity of DKDp189 for response to anti-hypertensive treatment, we performed a subset analysis in the DIRECT-Protec 2 cohort of patients who did not receive anti-hypertensive treatment. As expected, we found no statistically significant differences in the model's predictive ability between uncontrolled and controlled patients in this cohort (AUC = 0.561, 95% CI 0.492–0.628, *P*-value = .1232).

Even though individual peptides are of very moderate use as single biomarkers, investigating their regulation may inform about molecular pathophysiology, linking DKD progression under RASi therapy to specific molecular processes. To streamline and focus the assessment, we investigated which 227 peptides showed consistent regulation in all three cohorts, and in DC-REN in all four eGFR percentage slope assessment approaches. This resulted in the identification of 92 peptides, listed in Supplementary data, Table S5. The most prominent finding is a reduction of 33 collagen alpha-1(I) (COL1A1) fragments (while only one COL1A1 fragment showed increased abundance) in uncontrolled patients, and a consistent increase of 4 alpha-1-antitrypsin fragments. Additionally, 41 collagen fragments were also consistently regulated; in most cases (*n* = 35) reduced, while only 6 collagen-derived peptides are found to be up-regulated.

## DISCUSSION

We had hypothesized that urinary proteomics can identify biomarker peptides able to predict the response to RASi treatment to avoid DKD progression, as defined by four different equations used to calculate eGFR.

The data presented in this manuscript have an impact in several areas. The two key findings are that the differences in eGFR based on different methods are minor in absolute values, as is well established; however, when investigating change in eGFR over time, which is the most widely used approach to assess CKD progression, results differ widely, depending on the method used. As such, the assignment of uncontrolled or controlled patients can only be made with moderate certainty. The second key finding is that specific urine peptides are associated with response (or lack thereof) to RASi treatment to reduce DKD progression.

Given the high prevalence of CKD in diabetic patients and the potential impact of accurate GFR estimates on clinical decision-making and patient outcomes, it is important to investigate the performance of the commonly used eGFR equations to correctly classify patients who will progress to DKD, as well as the prognostic ability in this specific population.

Our analyses demonstrate that patient classification by the different equations varied considerably among them when investigating change in eGFR. This suggests that the choice of method/equation could impact patient stratification and subsequent clinical management, in concordance with previous studies demonstrating that the prevalence of CKD differed depending on the equation used [16, 18, 29–33].

Furthermore, we demonstrate that the different equations result in the definition of distinctly different potential biomarkers. Notably, the EKFC eGFRcys equation demonstrated the highest number of peptides verifiable in the test cohorts and strongly correlated with the peptides identified by the other methods. Previous studies have shown that cystatin C levels are less affected by some clinical factors [14, 34]. Moreover, the EKFC eGFRcys study equation demonstrated that this provided unbiased results and improved the accuracy of GFR assessment over commonly used equations, and was even less dependent on certain demographic parameters [15].

Most of the peptides associated with the progression of CKD are COL1A1 fragments, which have been identified as components of diagnostic models in different studies of CKD [35–37]⁠. These peptides are often decreased in patients with progressive CKD [38, 39]. Previous studies have reported that the normal tissue repair process in the kidney is deregulated in progressive patients, resulting in pathological accumulation of extracellular matrix (ECM) proteins such as collagens, mainly type I and III, leading to kidney fibrosis [8]. This suggests a disbalance between the accumulation of collagens and the decrease in peptide levels, indicating both a reduction of collagen degradation and an increase in collagen synthesis [11, 38].

Furthermore, we observed a consistent and significant change in the abundance of several peptides that have been previously associated with the progression of kidney diseases, including alpha-1-antitrypsin, antithrombin-III (ATIII), CD99 antigen and uromodulin [35, 40, 41]. Alpha-1-antitrypsin is a hepatic stress protein and protease inhibitor that has been suggested as a biomarker for kidney damage [42]. High levels of alpha-1-antitrypsin could potentially be associated with the inhibition of collagenase activity, which is compatible with the decrease in collagen fragments observed in patients with CKD [11]. ATIII protein has been considered a potential noninvasive biomarker in CKD due to its role as a serine protease inhibitor in the coagulation cascade and its involvement in the inflammatory process [43, 44]. Previous studies have demonstrated that low levels of ATIII are associated with an increased risk of thrombosis and reduced anti-inflammatory effects, which can contribute to the progression of kidney diseases [43, 45]. Our results revealed increased peptide levels, suggesting an increase in the degradation of ATIII.

In addition, we consistently observed reduced levels of CD99 antigen peptide in uncontrolled patients. CD99 antigen is a transmembrane glycoprotein that plays a crucial role in inflammation, cell survival, proliferation, differentiation and stress response [46]. Moreover, it is involved in cell adhesion processes among different types of cells and ECM components. Recently, deregulation of CD99 antigen peptides were implicated in COVID-19-associated kidney injury and CKD in different studies [12, 41, 47].

Urinary peptide panels have been successfully applied to determine the prognosis and the risk of mortality in CKD patients [12, 36]. In this study, we observed significant variability in outcomes of various eGFR equations on patients stratification as uncontrolled or controlled. This presents a challenge for the development of a universally applicable classifier. Furthermore, we selected the most frequently concordant differential peptides (*n* = 189) to develop a urinary biomarker-based model to predict DKD progression. The DKDp189 model showed significant differences between patients with uncontrolled and controlled DKD. The progression definition was established using rigorous criteria to ensure that the cases and controls had well-defined phenotypes, increasing the likelihood of identifying valid predictive biomarkers. Furthermore, many of the peptides identified have been associated with pathological processes related to CKD progression.

Although the AUC in the validation cohorts was not high, the significant differences observed between uncontrolled and controlled kidney function in the model highlight the potential used of this peptide panel as prognostic tools in DKD patients. Additionally, to assess the predictive value of the model with respect to albuminuria and the CKD273 model, we performed logistic regression analysis in the controlled and uncontrolled patients of the different cohorts. As expected, we did not observe additional predictive value of albuminuria or CKD273 in patients treated with RASi (Supplementary data, Table S6).

This study has several limitations. First, it is a retrospective observational study. However, we have investigated additional datasets from two well controlled prospective studies to estimate the prognostic value of the newly developed model. Second, we only had creatinine-based eGFR values in the two independent validation cohorts. However, this shortcoming is expected to, in fact, harm the performance of the model by increasing variability; hence the true performance (assessed based on cystatin C measurements) is expected to be even better. Moreover, the new CKD-EPI equations [48] were not taken into account. This decision was made because the data were collected from central European studies, and including additional eGFR values from these GFR equations may not have been relevant within the specific context of this study. The number of subjects may also limit the generalization of the findings; consequently further studies are necessary, in larger cohorts.

Collectively, the DKDp189 model demonstrates potential as a predictive tool for guiding treatment with RASi in diabetic patients. Future research should include a more extensive study population to validate further and refine the DKDp189 model and explore its utility in other CKD populations.

## DATA AVAILABILITY STATEMENT

Anonymized data used in conducting the analyses will be made available upon request directed to the corresponding author. Proposals will be reviewed and approved by the authors with scientific merit and feasibility as the criteria. After approval of a proposal, data can be shared via a secure online platform after signing a data access and confidentiality agreement. Data will be made available for a maximum of 5 years after a data sharing agreement has been signed.

## Supplementary Material

gfad223_Supplemental_Files
