# Supplementary figures and images for: Urinary peptide analysis to predict the response to blood pressure medication
Source: Nephrol Dial Transplant. 2023 Oct 31;39(5):873–83. doi: 10.1093/ndt/gfad223 (PMC11181870; doi:10.1093/ndt/gfad223)

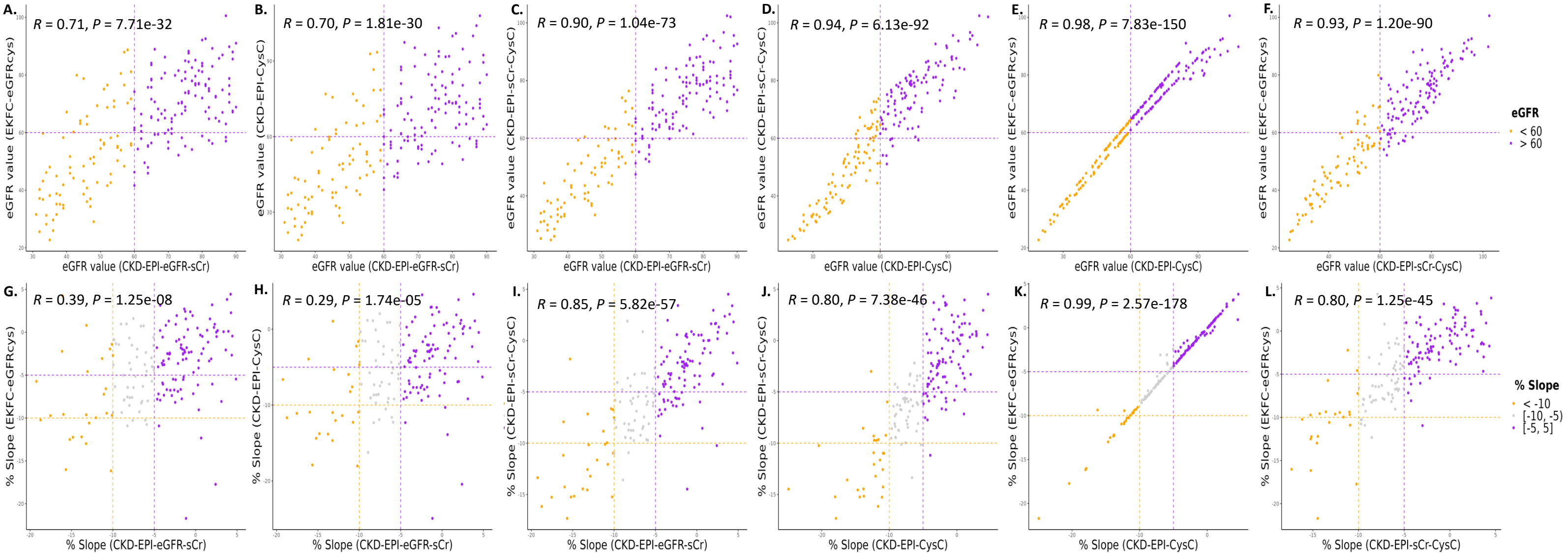

Supplement: gfad223_Supplemental_Files [file gfad223_Supplemental_Files.zip › SupplementaryFigure1. Association of baseline eGFR and slope_allequations.pdf]
